# Diagnosing a wide complex tachycardia using basic electrophysiologic properties of the cardiac conduction system

**DOI:** 10.1002/joa3.12657

**Published:** 2021-11-23

**Authors:** Gregory P. Siroky, Davendra Mehta

**Affiliations:** ^1^ Department of Cardiology Division of Electrophysiology Mount Sinai Morningside Icahn School of Medicine at Mount Sinai New York New York USA

**Keywords:** atrial tachycardia, electrocardiogram, linking, Mobitz I AV block, phase 3 block

## Abstract

65‐year‐old man with a history of coronary artery disease s/p percutaneous coronary intervention to the left anterior descending artery and atrial fibrillation s/p recent (<3 months) pulmonary vein isolation presented to the emergency department with symptoms of palpitations for 1 day after admittedly forgetting to take his medications found to be in a wide complex tachycardia. We discuss a stepwise approach using properties of the conduction system to diagnose the patient’s tachycardia.

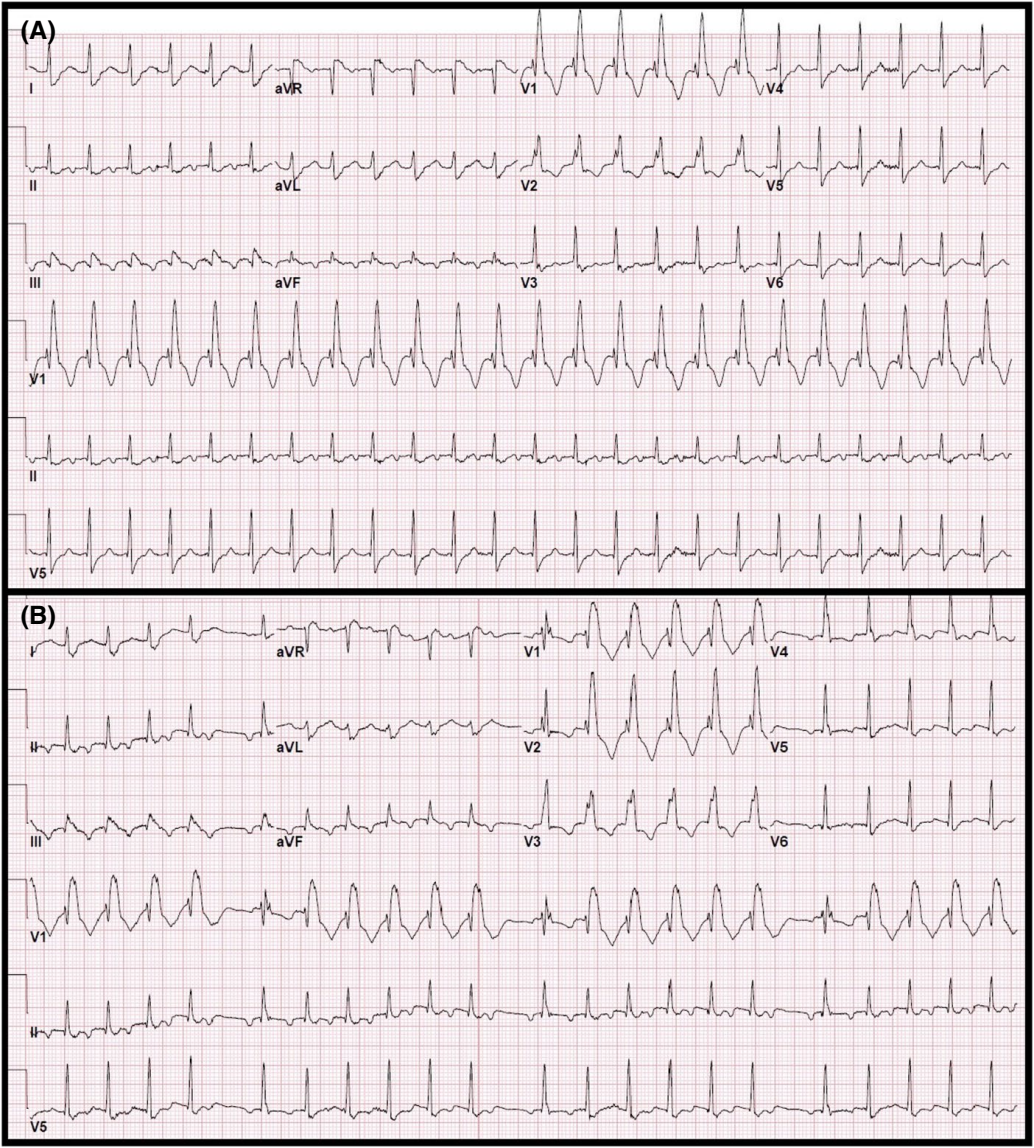

## 1 CASE

**FIGURE 1:**
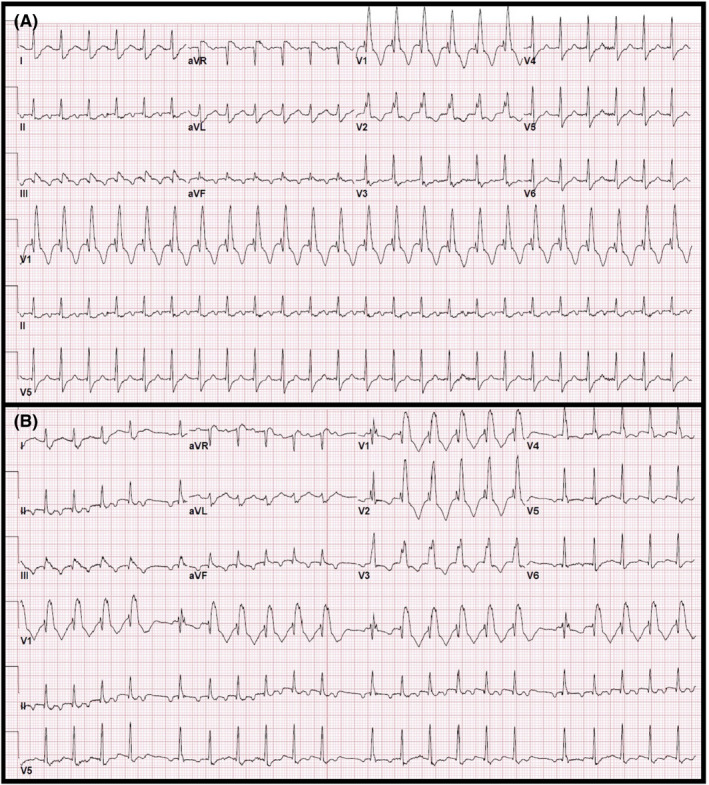
(A) Presenting ECG. (B) ECG obtained 10 min after administration of IV metoprolol

A 65‐year‐old man with a history of coronary artery disease (CAD) status post percutaneous coronary intervention (PCI) to the left anterior descending artery (LAD) and atrial fibrillation (AF) status post recent (<3 months) pulmonary vein isolation (PVI) presented to the emergency department with symptoms of palpitations for 1 day after admittedly forgetting to take his medications. The presenting ECG is shown in Figure 1A. The patient's BP was stable at 110/70 mm Hg and laboratory results were unremarkable including serum potassium (K) and magnesium (Mg) levels of 4.2 mmol/L and 2.2 mmol/L, respectively. Bedside echocardiogram demonstrated an ejection fraction of 45% with anterior wall hypokinesis. Administration of 6mg, followed shortly by 12mg, of adenosine had no effect, however, an ECG obtained 10 min after intravenous (IV) metoprolol is shown in Figure 1B. What is the differential diagnosis of the ECG shown in Figure 1A? How does the ECG in Figure 1B aid in determining the diagnosis?

## 2 COMMENTARY

**FIGURE 2:**
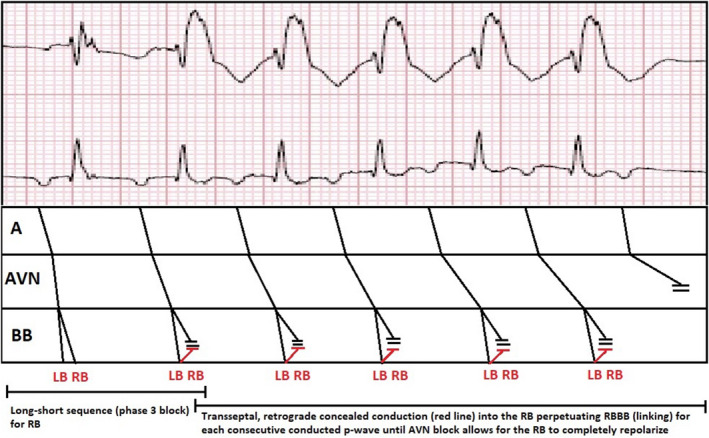
Ladder diagram demonstrating the Mobitz I AV block and phase 3 block of the RB with perpetuation of RBBB due to transseptal, retrograde concealed conduction (linking). A, atrium; AVN, atrioventricular node; BB, bundle branches; LB, left bundle; RB, right bundle

The ECG in Figure 1A shows a regular, wide complex tachycardia (WCT) at a rate of 150 bpm with right bundle branch block (RBBB) morphology (QRS duration of 140 ms) and borderline right axis deviation and as well as a 1:1 atrial (A) to ventricular (V) ratio. A broad differential includes supraventricular tachycardia (SVT) with aberrancy versus ventricular tachycardia (VT). While a left anterior fascicular VT could be a possible diagnosis given the RBBB/left posterior fascicular block (LPFB) pattern, the ECG in Figure 1B demonstrates that the A:V ratio is greater than 1, therefore excluding VT. A double tachycardia in which an atrial tachycardia (AT) and VT occur at the same time is possible, however, it is rare and unlikely given the atrial and ventricular rates in Figure 1A are the same and the grouped beating in Figure 1B rule this out. Possible SVT mechanisms of a mid to long RP tachycardia include sinus tachycardia (ST), atrial flutter (AFL), atypical atrioventricular nodal reentrant tachycardia (AVNRT), atrioventricular reciprocating tachycardia (AVRT) using a right sided, decremental posteroseptal accessory pathway (AP), or AT. A stepwise approach must be undertaken by interpreting the pathology/physiology of each part of the conduction system starting with the sinus node/atrium, followed by the AV node, and then the His‐purkinje system. Organized atrial activity at 150bpm is seen before each QRS (which is more evident in Figure 1B) and determining whether the atrial activity is emanating from the sinus node versus an ectopic focus is dependent on the p‐wave axis. P‐waves are inverted in the inferior leads as well as in lead V1 making this a low right atrial activation and ruling out sinus tachycardia. In addition, due to an atrial rate of 150 bpm, it is unlikely to be AFL. Next, the lead II rhythm strip (Figure 1B) shows grouped beating with each QRS associated with a preceding p‐wave and prolonging PR segment thereby diagnosing 7:6 s degree, Mobitz I AV block. Due to an obligatory 1:1 AV relationship, AVRT is excluded. Of the remaining possible SVTs, AVNRT with 7:6 Mobitz I and lower common final pathway block is possible,^1^ however, AT with Mobitz I block is the more likely diagnosis. Understanding the mechanism of the grouped WCT (Figure 1B), which is the same morphology as in Figure 1A, requires basic knowledge of the electrophysiologic properties of the His‐purkinje system and its respective action potentials. Due to the long‐short sequence following the last non‐conducted p‐wave in each group, when the second p‐wave in the sequence conducts down the AV node it falls on phase 3 of the right bundle branch action potential rendering it refractory and thus leading to a right bundle branch block (RBBB). The RBBB persists due to transseptal, retrograde concealment into the right bundle, or linking, until the last p‐wave in the sequence does not conduct and allows the right bundle to repolarize (Figure 2). Fortunately, this is a physiologic phenomenon and further confirms that the WCT in Figure 1A is aberrant conduction and not VT. Therefore, the final interpretation of the ECGs shown in Figure 1A and 1B are AT with RBBB aberrancy and AT with Mobitz I AV block and phase 3‐induced right bundle branch block with linking, respectively.

The most challenging part of interpreting a WCT is determining whether the mechanism is due to aberrant conduction versus VT, especially in light of a history of prior myocardial infarction. While there are numerous algorithms to help differentiate between the two,^2^ a basic understanding of basic physiology is all that is needed in this case. VT was excluded quickly as the patient's post‐metoprolol ECG demonstrated an A:V ratio of greater than 1, leaving aberrant conduction as the cause of WCT. In addition, the patient's history of recent AF ablation was key in diagnosing the arrhythmia, as the incidence of AT post AF ablation is 5 Mobitz 40%.^2^, ^3^ There are various causes of aberrated conduction: (1) pre‐existing and/or physiologic/functional bundle branch or interventricular conduction block, (2) metabolic derangements (i.e., hyperkalemia), and (3) drug‐induced (i.e., propafenone toxicity).^4^ The described mechanisms of functional/physiologic block include acceleration‐dependent block above the critical heart rate, phase 3 block of the action potential, phase 4 bradycardia‐dependent block due to disease in the His‐Purkinje system, and transseptal, retrograde, concealed invasion of the bundle branch (BB) rendering it refractory to subsequent depolarizations.^5^ Our patient's ECG (Figure 1B) demonstrates two of these mechanisms: phase 3 block, which caused the first aberrated QRS in the group followed by transseptal, retrograde, concealment into the RB perpetuating the aberrancy.

Shortly after the ECG in Figure 1A was taken, the patient self‐converted to sinus rhythm and as he was still within the 3‐month blanking period post ablation, a repeat ablation was not offered. Fortunately, a one‐month event monitor did not demonstrate any further arrhythmias and the patient had been symptom free.

## CONFLICT OF INTEREST

The authors have nothing to disclose.
